# Blocking IL-1 to prevent respiratory failure in COVID-19

**DOI:** 10.1186/s13054-020-03166-0

**Published:** 2020-07-18

**Authors:** Frank L. van de Veerdonk, Mihai G. Netea

**Affiliations:** 1 Department of Internal Medicine and Radboud Center for Infectious Diseases, Radboud University Medical Center, 6500HB Nijmegen, The Netherlands; 2 Immunology and Metabolism, Life & Medical Sciences Institute, University of Bonn, 53115 Bonn, Germany

## Abstract

COVID-19 is an emerging disease that can manifest itself as asymptomatic or mild respiratory tract infection in the majority of individuals, but in some, it can progress into severe pneumonia and acute respiratory distress syndrome (ARDS). Inflammation is known to play a crucial role in the pathogenesis of severe infections and ARDS and evidence is emerging that the IL-1/IL-6 pathway is highly upregulated in patients with severe disease. These findings open new avenues for host-directed therapies in patients with symptomatic SARS-CoV-2 infection and might in addition to antiviral treatment be enough to curb the currently unacceptably high morbidity and mortality associated with COVID-19.

## Introduction

Although the majority of patients with COVID-19 are asymptomatic or have mild SARS-CoV-2 infections, many patients have been hospitalized and admitted to intensive cares (ICUs) and mortality is significant. Understanding this outbreak, including the effectiveness of supportive, immune-modulatory, and antiviral treatments, is essential. An important aspect of severe COVID-19 is a hyperinflammatory status, and immunomodulatory therapy might therefore be an important aspect in the treatment of COVID-19. Although ICU patients have been treated with glucocorticoids, some experts have even argued, based on studies in Middle-Eastern respiratory syndrome coronavirus (MERS-CoV), severe acute respiratory syndrome (SARS), influenza, and respiratory syncytial virus (RSV), that they are likely to do more harm than good [1, 2]. However, a more recent study showed that early short-course corticosteroids during admission were associated with fewer ICU admissions [3], and a yet to be published study on dexamethasone argues that it can save lives especially in mechanically ventilated patients. Other immune modulatory treatments of interest include blocking the IL-1 or IL-6 pathway, the use of interferon-β, and many others. There are currently only small observational trials that contribute to the evidence for the benefit or harm of these interventions in COVID-19. In addition to the lack of available treatments known to be effective, insight into the pathophysiology of this coronavirus needs to be urgently addressed. This is essential in the pathway towards developing new, or repurposing existing, therapies that can be used in the treatment of patients with SARS-CoV-2.

## Mechanism of disease

The novel coronavirus SARS-CoV-2 is highly likely to have similarities to other coronaviruses such as SARS-CoV, with which it has the strongest sequencing similarities, and other severe respiratory virus infections such as *influenza*. The most severely ill patients infected with SARS-CoV or influenza seem to develop an immune phenotype that can be described as an inflammasome-mediated hyperinflammatory status, causing respiratory failure and secondary infections [4–9]. Autopsy in young, previously healthy, patients that died during the H1N1 influenza 2009 pandemic revealed evidence of “cytophagocytosis” in the lung [10]. This phenomenon is a hallmark of macrophage activation syndrome (MAS), or named secondary HLH (sHLH) [11, 12]. Lethal complications of influenza are consistent with inflammasome-mediated disease with signs of MAS [11, 13–15]. Inflammasomes are protein complexes that activate caspase-1 protease that in turn processes proinflammatory cytokines from the IL-1 family (e.g., IL-1β, IL-18) into active cytokines. A whole-exome study performed in fatal cases of H1N1 with signs of MAS found a high percentage of mutations in genes that are linked to genetic causes of diseases similar to MAS/HLH, suggesting that the genetic background of the patient predisposes to developing MAS in influenza [16]. Underscoring the importance of the inflammasome/IL-1 pathway in MAS is the observation that a monogenetic mutation in the inflammasome underlies primary MAS [17].

In the case series of critically ill patients with SARS from Toronto and Singapore, ARDS and multiple organ failure were frequently observed [8, 18]. ARDS was thought to be due to an exacerbated innate host response to SARS-CoV [19, 20]. Similar pulmonary hyperinflammation was seen on the histology in MERS patients [21]. Lung histology in COVID-19 shares similarities to SARS, MERS, and influenza [22]. Another study revealed high IL-18-circulating concentrations [23], a cytokine that is associated with MAS [24], and MAS has also been reported in SARS [25]. In SARS-CoV-2 infection, elevated IL6 and ferritin concentrations have also been described [26]. In other studies with SARS-CoV-2 pneumonia, patients that needed ICU admission more often had leukocytosis, higher neutrophil counts, lower lymphocyte counts, elevated D-dimer, and highly elevated LDH, and the main reason patients were admitted to the ICU was ARDS [27–29].

However, currently, it becomes clear that only a minority of patients with COVID-19 develop MAS/HLH. Many other patients show signs of a cytokine storm syndrome, but do not fulfill the criteria of MAS/HLH. We and others have identified an IL-1/IL-6-driven innate immune response [30–36]. Interestingly, TNF concentrations in plasma were not significantly elevated in the early stages of disease in critically ill patients compared to patients admitted to the ward: median 24.0 pg/ml [IQR 16.5–33.5] and 21.5 pg/ml [IQR 16.0–33.5], respectively [30]. This might explain that hemodynamic instability is not a classical presentation of COVID-19, since both TNF and IL-1 as cytokines are needed to cause hemodynamic problems [37], arguing that the hyperinflammatory innate immune response initially is mainly an overactive IL-1/IL-6 response. Also, the classical coagulopathy associated with COVID-19 is different by not fulfilling the criteria of classical diffuse intravascular coagulopathy (DIC), despite D-dimers being profoundly elevated in many critically ill patients [38]. This all points to a unique underlying pathophysiology in COVID-19 that might be partially explained by the local ACE2 deficiency in the lung and subsequent effects on the kallikrein-kinin system [39]. Next to inflammation and coagulopathy, COVID-19 has a clear vascular component with an “endothelitis” that has been observed in the lungs of patients that died [22, 40]. These are unique features of the disease, underscoring that SARS-CoV-2 can infect and inflame endothelial cells and make blood vessels leak.

## Targeting IL-1 in COVID-19

**Fig. 1:**
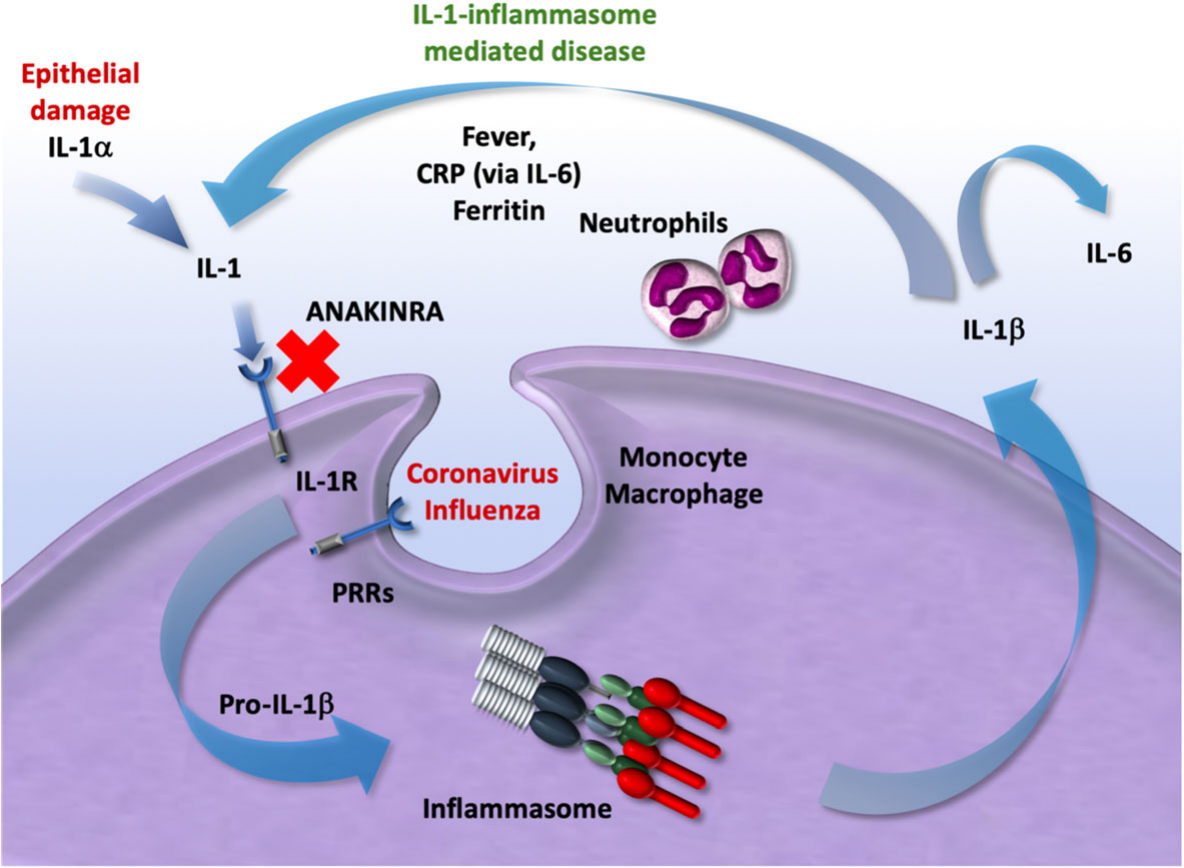
Rationale for use of anakinra in severe coronavirus. The SARS-Cov-2 will cause epithelial damage leading to the release of IL-1α that will (1) recruit neutrophils and monocytes to the site of infection and (2) induce IL-1β in monocyte/macrophages. Moreover, the 2019 nCov will induce pro-IL-1β in monocyte/macrophages which in turn will induce more IL-1 that will recruit and activate more innate immune cells. This autoinflammatory loop where IL-1 (IL-1α and IL-1β) can induce production and release of more IL-1 has to be tightly regulated because an ongoing loop will activate and recruit more innate immune cells independent of the initial trigger. Anakinra blocks the IL-1 receptor (IL-1R) and thus will prevent autoinflammation by blocking effects of IL-1α released from dead epithelial cells, as well as IL-1β produced by immune cells. IL-1-induced IL-6 will also be blocked. The autoinflammatory loop can exacerbate from increase innate immune response into uncontrolled MAS a spectrum that associates with increasing ferritin levels

Clinical and laboratory features of MAS include sustained fever, hyperferritinemia, and high IL-18 concentration in circulation, pancytopenia, fibrinolytic consumptive coagulopathy, and liver dysfunction [11, 12, 24]. Controlling this excessive immune activation and organ damage can be achieved in various ways. With MAS in the context of other triggers, the focus of treatment is on interrupting the cytokine storm, because cytokines such as IL-1 maintain the persistent drive of inflammasome activation (Fig. 1). IL-1 can induce production and release of more IL-1, a process described as an autoinflammatory loop (Fig. 1). Breaking this loop can be done by corticosteroids or chemotherapy and has been suggested for H5N1 infection [41]. However, these treatment strategies will also impair host defense against bacteria and fungi and will make patients prone to secondary infections that are a major cause of death of complicated viral pneumonitis [4, 6]. One of the safest ways to stop this overwhelming innate immune response can be accomplished by using IL-1 receptor (IL-1R) blockade or drugs that target IL-1 signaling [42, 43]. This approach, especially for the treatment of secondary MAS without underlying cancer, has recently been reported [44]. Anakinra is a bio-engineered form of the naturally occurring interleukin-1 receptor antagonist (IL-1ra) that blocks the action of interleukin-1 (Fig. 1). It is routinely used in patients with autoimmune and inflammatory disorders and MAS [44]. Anakinra has been used in several studies for sepsis and septic shock. Studies that recruited in total almost 2000 patients demonstrated that although anakinra did not reduce the overall all-cause mortality, survival was increased in the subgroup of sepsis patients with features of MAS (ferritin elevations in excess of 2000 ng/ml, coagulopathy, and liver enzyme elevations) [45–48]. Its safety profile and wide therapeutic margin on the one hand, and the central role of IL-1 in the cytokine storm of MAS on the other hand, warrants assessing anakinra as a potential therapeutic in severe coronavirus infection [49]. In tracking the effectiveness of the treatment, ferritin and IL-18 circulating concentrations are accepted biomarkers. Ferritin is an established laboratory test that is available in almost all hospitals in the Western world and can thus provide a simple and rationale biomarker for the development and resolution of MAS and can be used to follow the effect of anakinra on MAS-like inflammation in COVID-19.

### Treat with anakinra, do not wait for full-blown sHLH

It has recently been suggested to be aware of MAS and use a Hscore to calculate and consider immunosuppressive treatment such as corticosteroids, anakinra, tocilizumab, or JAKinibs [50]. A recent study showed that treating patients with COVID-19 that fulfilled these criteria with anakinra showed a beneficial response compared to historical outcomes [51]. However, three patients out of 8 died in spite of treatment, and patients who full-fill these criteria are likely to be so severely ill that any single intervention has a very marginal chance to drastically improve the outcome. In addition, a case series of 3 patients with leukemia were treated with anakinra based on extreme ferritin levels and hyperinflammatory status with beneficial clinical effects [52], and a case series of 5 patients with severe COVID-19 also showed a response to anakinra [53]. We propose to use immunomodulatory therapy with anakinra in an earlier phase then after admission on the ICU. First, the start of early treatment will prevent sHLH instead of treating it, which gives a better chance to the patient to survive this very severe complication. Second, anakinra has only mild immunosuppressive effects since it does not decrease the capacity to clear bacterial or fungal infections, and there are even data to support the assumption that blocking IL-1 might increase certain components of dysregulated host defense [54, 55]. Moreover, in contrast to JAKinibs, anakinra will not directly block the IFN-STAT1/STAT2 pathway critical for host defense against viral infections. Third, in contrast to tocilizumab (an IL-6 inhibitor), it targets and inhibits the core mechanism in the pathogenesis of MAS, namely the hyperactive inflammasome loop (Fig. 1). In addition, anakinra will decrease IL-6 production since IL-1 is a potent inducer of IL-6, and thus, the suggested beneficial effects of tocilizumab are likely to be seen also in anakinra. Anakinra will not only block IL-1β but also IL-1α which is released due to epithelial and endothelial damage and, in this way, targets the tissue-driven inflammatory response. Finally, the safety profile of anakinra is very good and the short half-life makes it possible to stop fast once undesired effects are seen such as neutropenia, which is not possible with tocilizumab. These arguments have led to the selection of anakinra as an immunomodulatory treatment option in several ongoing trials. A recent and larger study supports the use of anakinra in COVID-19 patients in the early phase and reports that high dose intravenous anakinra started in patients outside of the ICU was safe and resulted in clinical benefit in 72% of patients [56]. Another recent COVID-19 study included 52 consecutive patients for anakinra treatment and 44 historical patients. Admission to the ICU for invasive mechanical ventilation or death occurred in 13 (25%) patients in the anakinra group and 32 (73%) patients in the historical group and the treatment effect of anakinra remained significant in the multivariate analysis [57].

## Conclusions

Treating patients that are critically ill during a pandemic with a novel pathogen is a major challenge. As long as we do not have a vaccine or effective antiviral drugs, we need other strategies to help patients with COVID-19. One strategy that is urgently needed is to prevent disease progression from symptomatic to ICU. In the ICU, COVID-19 has many features including thromboembolic events, fibrosis, and “endothelitis,” conditions that are all difficult to treat. We propose that targeting the innate inflammatory response with anakinra in combination with supportive care and the best antiviral available could result in a drastic decrease of ICU admissions. One pitfall to date could be that using anakinra only in sHLH as proposed [50] might fail to help patients because sHLH is already associated with a high mortality despite treatment. We propose that when a clinician considers to dampen hyperinflammation with anakinra, this should be done on the ward or at entry of ICU due to several strong arguments: its safety profile and clinical experience in sepsis, the rationale to prevent full-blown sHLH, and the possibility to stop the drug without having undesirable long-term effects of anakinra.

## Data Availability

Not applicable.
